# A Case Report of Incidental Thyroid Cancer Metastasis Detected in a PSMA PET/CT Scan of a Prostate Cancer Patient: Predicting RAI-Refractoriness

**DOI:** 10.1055/s-0045-1812053

**Published:** 2025-10-09

**Authors:** Isavelina F. C. Gries, Eduardo E. S. Ongkeko, Patrick E. A. Fernando

**Affiliations:** 1 Department of Nuclear Medicine and Theranostics, St. Luke's Medical Center, Global City, Metro Manila, Philippines

**Keywords:** PSMA metastatic thyroid cancer, differentiated thyroid carcinoma, radioactive iodine-refractory, RAIR-DTC

## Abstract

Early recognition of patients at risk for radioactive iodine refractoriness (RAIR) is essential, as these individuals often face a poorer prognosis. Consequently, there is a pressing need for advanced therapeutic options in aggressive thyroid cancer cases. We present a case involving a 57-year-old male with prostate adenocarcinoma who, through prostate-specific membrane antigen (PSMA) PET/CT scanning, was incidentally found to have a PSMA-avid lung nodule. Subsequent biopsy confirmed metastatic thyroid carcinoma. Following two sessions of radioactive iodine (RAI) therapy, posttherapy scans showed a noniodine-avid lung nodule. The PSMA-avid lung lesion indicates an aggressive disease form likely to develop RAI resistance, which was subsequently confirmed. This finding suggests that PSMA expression could be an effective early predictor of RAIR. Moreover, the PSMA avidity of these lesions supports the use of Lutetium-177-PSMA therapy, expanding treatment options for RAIR-differentiated thyroid carcinoma (DTC).

## Introduction

Differentiated thyroid carcinoma (DTC) in Filipinos is linked to a more aggressive recurring behavior, leading to higher morbidity rates. 1 It is crucial to be vigilant and improve the quality of diagnosis, treatment, and prognosis of thyroid cancers in the Philippines. With the advent of prostate-specific membrane antigen (PSMA) PET/CT, multiple studies have described incidental uptake in the thyroid gland. Such findings may represent both benign and malignant etiologies, which warrant further clinical investigation. Presently, there is limited evidence on the potential of PSMA PET/CT scans as a prognostic biomarker in DTC, and opinions on their use in thyroid cancers, especially compared with ^18^ F-FDG PET/CT scans, are varied. 2 3 4 We aim to highlight the significance of simultaneous cancers identified by PSMA PET/CT scan, examine its predictive value in radioactive iodine refractoriness (RAIR)-DTC, and explore how PSMA radioligand therapy can enhance treatment protocols in the future.

## Case Report

**Fig. 1:**
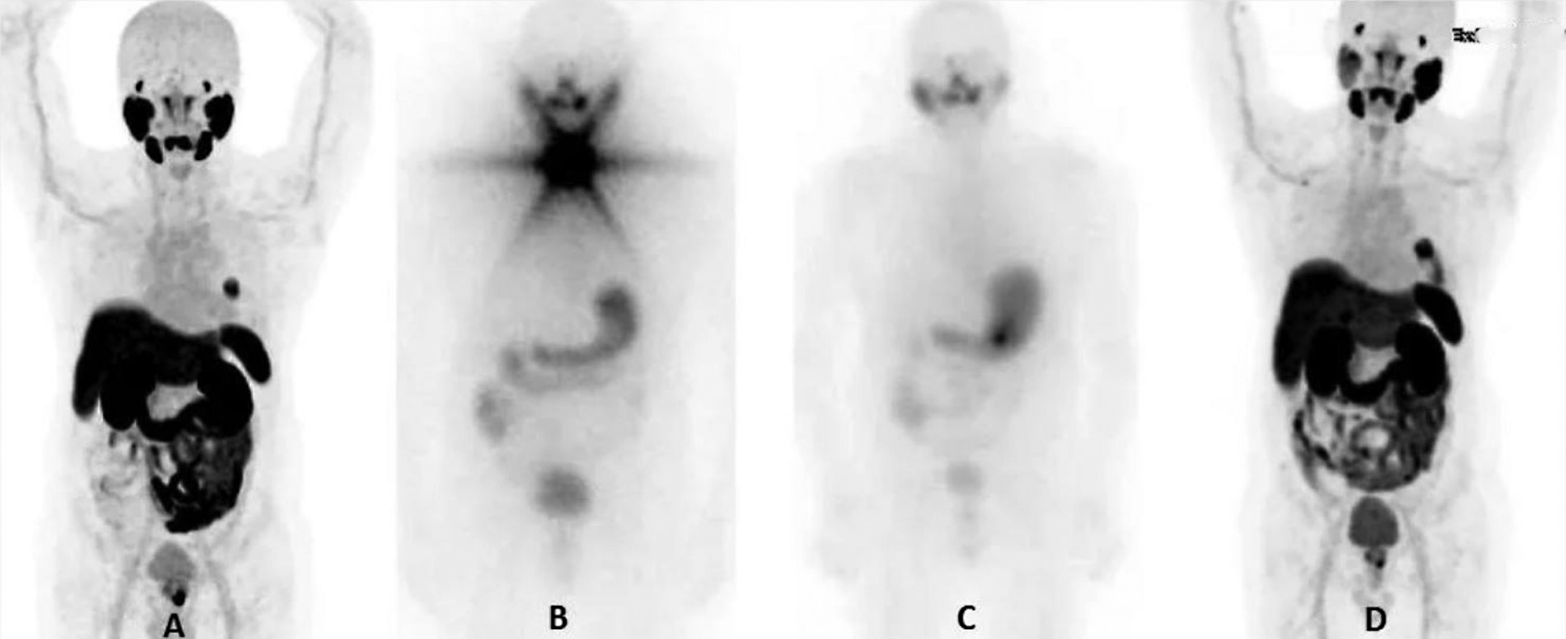
( **A** ) PSMA receptor-overexpressing lesions in the prostate and left lower lung nodule. ( **B** ) Posttherapy scan after first cycle of RAI therapy showed functioning thyroid tissue remnants in the anterior neck with a noniodine-avid left lung nodule. ( **C** ) Posttherapy scan after the second cycle of RAI therapy showed successful ablation of the neck remnants and persistent nonradioiodine concentration of the left lung nodule. ( **D** ) Interval decrease of PSMA receptor-overexpressing lesions in the prostate, while stable PSMA uptake in the lung nodule.

A 57-year-old male, diagnosed with prostate adenocarcinoma (Gleason score: 4 + 3 = 7, ISUP group 3), underwent an ^18^ F-PSMA PET/CT ( Fig. 1A ), which revealed PSMA receptor-overexpressing lesions in the prostate and left lower lung nodule, measuring 2.5 cm × 2.1 cm × 2.0 cm. Biopsy of the lung nodule, along with histomorphology and immunohistochemical staining, was consistent with metastatic thyroid carcinoma. Total thyroidectomy subsequently confirmed papillary thyroid microcarcinoma, and the patient subsequently underwent RAI therapy. Post-therapy total body scan ( Fig. 1B ) demonstrated functioning thyroid tissue remnants in the anterior neck with a noniodine-avid left lung nodule. A significant rise in serum thyroglobulin levels (26.82–561 ng/mL over a 4-month period) prompted a second cycle of RAI therapy, which achieved successful ablation of the thyroid remnants; however, there was persistent nonradioiodine concentration in the known lung metastasis ( Fig. 1C ). A follow-up ^18^ F-PSMA PET/CT (4 months since the first scan; Fig. 1D ) showed decreased PSMA uptake in the prostate and stable PSMA uptake in the lungs, with interval progression in size and number on CT. The patient is maintained on Pamorelin and has been initiated on Lenvatinib.

## Discussion

The PSMA-avid nodule in the left lower lung is attributed to increased PSMA expression in tumor-associated neovasculature, a phenomenon seen in aggressive and metastatic thyroid cancers, particularly in poorly differentiated subtypes. This expression is driven by angiogenic factors within the tumor microenvironment. 5 It is important to avoid assuming all PSMA-avid lesions in prostate cancer patients are metastases. In this case, biopsy of the PSMA-avid lung nodule revealed a synchronous papillary thyroid malignancy.

With the advent of PSMA PET/CT, several studies have reported incidental uptake in the thyroid gland. Although the PSMA PET/CT identified a metastatic lung nodule originating from the thyroid, it did not show PSMA uptake in the thyroid gland itself.

RAIR-DTC lacks the sodium iodide symporter in the basement membrane of thyroid follicular cells for iodine uptake. This absence explains why the lung nodule remains noniodine avid, despite receiving two cycles of radioactive iodine (RAI) therapy with a cumulative dose of 305.3 mCi. Identifying patients who are likely to develop RAI refractoriness is crucial, as these individuals have a 10-year survival rate of under 10%.

Emerging evidence indicates that PSMA expression may serve as a predictive biomarker for the early identification of RAIR-DTC. Immunohistochemical analyses have demonstrated upregulated PSMA expression within the tumor-associated neovasculature of RAIR lesions, a feature that correlates with increased tumor aggressiveness. Moderate to intense PSMA immunoreactivity has been significantly associated with an elevated likelihood of progression to radioiodine resistance. 6 7 8 In this case, PSMA-avid pulmonary metastasis was identified on PSMA PET/CT imaging prior to definitive biochemical or imaging evidence of RAIR, supporting the utility of PSMA expression as an early surrogate marker of refractoriness in DTC.

Furthermore, there is limited evidence on the potential of PSMA PET/CT scans as a prognostic biomarker in DTC. 2 3 4 It has been demonstrated that PSMA expression is related to malignant disease, poor prognostic factors, poorer progression-free survival, and a low rate of recurrence-free survival. 9 10

Due to the PSMA avidity of these metastatic lesions, therapy with Lutetium-177-PSMA has become a viable option, expanding treatment possibilities for RAIR-DTC. 11 Diagnostic PSMA PET/CT scans offer several added benefits, including detecting lesions with aggressive characteristics, early identification of radioiodine refractoriness, evaluating PSMA uptake in metastatic lesions, and assessing suitability for theranostic therapy. This case indicates that a PSMA PET/CT scan may be able to detect radioiodine resistance at an early stage and stratify patients in need of close monitoring, further evaluation, and other treatment options.

## Conclusion

This case indicates that PSMA PET/CT scans may be able to detect radioiodine resistance at an early stage and stratify patients in need of close monitoring, further evaluation, and other treatment options.
